# Toxoplasma, Toxocara and Tuberculosis co-infection in a four year old child

**DOI:** 10.1186/1471-2431-11-44

**Published:** 2011-05-26

**Authors:** Randeewari Guneratne, Devan Mendis, Tharaka Bandara, Sumadhya Deepika Fernando

**Affiliations:** 1 Intern House Officer, Colombo South Teaching Hospital, Kalubowila, Sri Lanka; 2 Consultant Paediatrician, Colombo South Teaching Hospital, Kalubowila, Sri Lanka; 3 Professor in Parasitology, Department of Parasitology, Faculty of Medicine, University of Colombo, Colombo, Sri Lanka

## Abstract

**Background:**

Tuberculosis. toxocariasis and toxoplasmosis are among the common infectious causes of lymphadenitis in children. Cases of *Toxoplasma gondii *and *Toxocara spp *co-infection have been reported.

**Case Presentation:**

This case report describes a co-infection of *Toxoplasma gondii*, *Toxocara *spp and tuberculosis in a child with chronic lymphadenopathy and eosinophilia.

**Conclusion:**

The case report highlights two important points. First is the diagnostic challenges that are encountered by clinicians in tropical countries such as Sri Lanka, where lymphadenopathy and eosinophilia with a positive serology commonly point towards a parasitic infection. Secondly the importance of proper history taking and performing the Mantoux test as a first line investigation in a country where the incidence of tuberculosis is low, even in the absence of a positive contact history.

## Background

Tuberculosis. toxocariasis and toxoplasmosis are among the common infectious causes of lymphadenitis in children [1]. Approximately 250,000 children worldwide develop tuberculosis, a larger proportion being reported from the South East Asian region [2,3]. Extra-pulmonary tuberculosis is more common in children, the most common form being lymphatic disease accounting for about two thirds of the cases of extra-pulmonary tuberculosis [4-6].

*Toxoplasma gondii *and *Toxocara *spp. infections are cosmopolitan zoonotic diseases which may cause systemic and ocular diseases in humans [7-9]. Few publications exist regarding *Toxoplasma *and *Toxocara *co-infection [10,11].

This case report describes a child with chronic lymphadenopathy and eosinophilia who was seropositive for both *Toxoplasma gondii *and *Toxocara *spp, together with a positive Mantoux test and lymph node histology suggesting tuberculosis.

## Case Presentation

A 4 year-old, previously healthy boy was admitted to the surgical unit of the Colombo South Teaching Hospital, Sri Lanka with an abscess in the left big toe. No fever or local lymphadenopathy was present at initial presentation. The abscess was drained, treated with antibiotics and the child was discharged. Two weeks later the child was re-admitted with an infection at the site of original abscess and left sided inguinal lymphadenopathy. Full blood count (FBC) revealed an eosinophilia of 12.5% (WBC count 19,800, N 40.8%, L 35.9%). Blood picture showed moderate eosinophilia with reactive changes suggestive of either a parasitic infection, or an allergic/drug reaction. A blood sample was sent for the detection of *Toxoplasma *and *Toxocara *antibodies to the Medical Research Institute (MRI), Colombo. Empirical treatment was commenced with Diethyl Carbamazine 6 mg/kg/body weight for 14 days and Mebendazole 50 mg twice a day for 3 days (based on body weight of 13 kg) together with intravenous antibiotics. The lymph node enlargement which persisted during the wound infection resolved with treatment and the child was discharged 14 days after admission.

Three months later the child was referred to the Paediatric Unit of the same hospital with reports of the blood sample taken at the time of previous admission indicating positive serology for both *Toxoplasma gondii *and *Toxocara *spp (*Toxoplasma *antibody IgG Negative, IgM Positive and *Toxocara *antibody IgG Positive). Examination of the child at this instance revealed bilateral cervical (2 cm) and left side inguinal (3 cm) lymph nodes, and non tender hepatomegaly approximately 2 cm from the costal margin. No splenomegaly was noted. There was no history of fever, cough, wheezing or recurrent infections. No weight loss, night sweats or chronic cough suggestive of tuberculosis was recorded. There was no contact history of tuberculosis though intensive questioning of the parents revealed a history of lymphadenopathy due to tuberculosis in the elder sibling, approximately one year before this child was born. The sibling had been treated with the full course of anti-tuberculosis treatment based on the WHO recommendations [12] and was healthy thereafter. There was no association with cats or ingestion of undercooked meat though there was a history of close contact with dogs which were not de-wormed.

The patient was re-admitted to the Paediatric Unit. Full blood count and blood picture was repeated and FBC showed a total count of 16,500 with 18.5% eosinophils (N 29%, L 46% M 5.4%). The blood picture was similar to the previous report. Ultrasound Scan abdomen confirmed mild hepatomegaly 3 cm from costal margin but no splenomegaly or para-aortic lymph node enlargement. As toxoplasmosis is generally a self- limiting disease in this age group, the child was treated with high doses of Albendazole (50 mg/kg per day in two divided doses to a maximum dose of 400 mg daily for 5 days) for toxocariasis.

Mantoux test was positive, suggesting co-existing tuberculosis in this child. Chest x- ray did not show any lesions suggestive of pulmonary tuberculosis. An inguinal lymph node biopsy was taken for histology on the 5^th ^day of treatment with Albendazole. A repeat FBC indicated that the blood counts were within normal range (Total count 10,800, N 47%, L 51%, M 1%, E 1%). However, the lymph node enlargement persisted.

The biopsy report of the left inguinal lymph node which was received two weeks later, indicated central necrosis with numerous tuberculoid type granulomata. Granulomata consisted epithelioid histiocytes. Langerhans type giant cells seen in central caseous necrosis. The appearance was compatible with tuberculous lymphadenitis with no evidence of *Toxoplasma *or *Toxocara *in the lymph node sample.

The parents were requested to take the child to the national chest clinic for anti tuberculosis treatment with instructions to report back to the Paediatric clinic with results of antibody tests for toxoplasmosis and toxocariasis both in the mother and child and an HIV Profile of the child. Acute *Toxoplasma *and *Toxocara *infection was confirmed in the child with positive anti *Toxoplasma *IgM and IgG antibodies and a four-fold rise in the IgG titre for toxocariasis as compared to the results taken 3 months previously justifying the treatment for toxocariasis. The HIV screening was negative. All test results were negative in the mother. The parents were requested to repeat the tests 6 months after completion of treatment and advice given to prevent re-infection of zoonotic parasitic diseases. They were also educated about tuberculosis and the importance of completing the full course of treatment.

## Conclusions

This case report highlights two important points. Firstly this child had a co-infection of toxocariasis, toxoplasmosis and tuberculosis and secondly diagnostic challenges were encountered by the clinicians as lymphadenopathy and eosinophilia commonly point towards a parasitic infection. The high eosinophil count with lymphadenopathy, positive serology and blood picture reports combined with a mild hepatomegaly could have limited the final diagnosis to *Toxoplasma, Toxocara *co-infection and the child may have been discharged following the appropriate treatment. This highlights the importance of proper history taking and performing the Mantoux test as a first line investigation even without a positive contact history in a country like Sri Lanka where the prevalence of tuberculosis is low [13]. As the risk of tuberculosis progression is high in very young children (<3 years), should the disease have not been detected the consequences may have been severe [14]. Transmission of tuberculosis occurred from an infectious person, possibly the elder sibling or a source in their community. Pinpointing the source of the tuberculosis infection may be particularly challenging in this case as the parents indicated that the sibling received full treatment at the time of diagnosis. Only genotyping would confirm whether this child and the sibling were infected with the same strain which is beyond the scope of this report.

The Medical Research Institute is one of the two government institutes in the country which carries out tests for anti *Toxocara *and *Toxplasma *antibodies free of charge. Results of repeat antibody tests carried out 3 months after the first test confirms an acute *Toxoplasma *and *Toxocara *infection. Seroprevalence of *Toxocariasis *in Sri Lanka is shown to be 43% in rural areas [15] and 20% in urban hospital population [16]. Human toxocariasis gives a diversity of clinical conditions ranging from non-specific covert toxocariasis to compartmentalized (ocular or neurological) toxocariasis [17]. Confirmation of these parasitic infections with investigations other than antibody tests proves difficult due to inadequate resources and the large patient numbers presenting to the state run institutes. Treatment is provided free of charge by the Government of Sri Lanka. As *Toxoplasma *causes a self- limiting infection in children no treatment was given. The child was treated for toxocariasis to prevent visceral migration of the parasite.

## Abbreviations

FBC: Full blood count; WBC: White blood cell count; N: Neutrophils; L: Lymphocytes; M: Monocytes; E: Eosinophils

## Competing interests

The authors declare that they have no competing interests.

## Authors' contributions

RW, DM and TB managed the patient during his stay in hospital and followed up on the patient there after. SDF was responsible for interpreting the results of the serological tests and drafting the manuscript. All authors read and approved the final manuscript.

## Pre-publication history

The pre-publication history for this paper can be accessed here:

http://www.biomedcentral.com/1471-2431/11/44/prepub
